# Successive Efficacy Evaluation of Various Commercial Live-Attenuated *Avian coronavirus* Vaccination Schedules Against a Local GI-23.3 Challenge in SPF Broilers

**DOI:** 10.3390/vaccines13111132

**Published:** 2025-11-02

**Authors:** Eman Abd ElMenum Shosha, Sara Abdelnaser, Ali Mahmoud Zanaty, Abd Elfattah ElZanaty, Karim Selim, Ibrahim Eldaghayes

**Affiliations:** 1 Virology Department, Faculty of Veterinary Medicine, New Valley University, El-Khargia 72511, Egypt; 2 Reference Laboratory for Quality Control on Poultry, Animal Health Research Institute (AHRI), Agriculture Research Center (ARC), Giza 12619, Egypt; 3 Poultry Disease Diagnosis and Research Department, Animal Health Research Institute (AHRI), Agricultural Research Center (ARC), Giza 12619, Egypt; 4 Department of Microbiology and Parasitology, Faculty of Veterinary Medicine, University of Tripoli, Tripoli P.O. Box 13662, Libya

**Keywords:** *Infectious bronchitis virus*, live attenuated vaccine, vaccine efficacy, GI-23, vaccination regimes

## Abstract

**Background:** *Infectious bronchitis virus* (IBV) is a highly spreading, evolving virus that induces multiple manifestations, including respiratory, urinary, and reproductive symptoms, and presents a considerable risk to the Egyptian poultry sector. This study assessed various IBV vaccination protocols available in broiler populations comprising live attenuated vaccines such as IB Var II, 793/B (4/91), IB Primer, and H120 against the local novel IBV-GI-23.3 strain. **Methods:** Vaccines were administered to eight groups of SPF chicks at 1 day only or 1 + 14 days of age. Birds were challenged via the oculo-nasal route at 28 days of age using 10^6^ EID_50_/0.2 mL/chick with the NewValley-1-EGYIBV-GI23.3-2023 local strain. Ciliostasis activity and the scores for histopathological lesions were evaluated at 7 days post-challenge (DPC). Virus shedding was monitored at 3, 5, and 7 DPC using the real-time RT-PCR method. **Results:** The ciliostasis test indicated that the vaccinated groups receiving the IB Primer + 4/91 vaccine regime at 1 day only or 1 + 14 days of age received the highest level of protection (65%, 68%, respectively). Similarly, administration of IB Primer + IB Var II at 1 + 14 days of age demonstrated substantial protection (63%). Conversely, administering the H120 + 4/91 vaccination protocol at days 1 and 14 resulted in a moderate level of protection (53%). Tracheal IBV shedding quantification and subsequent histopathological signs of trachea, proventriculus, bursa, and kidney degenerative changes were significantly lower in the vaccinated groups (especially the IB Primer + 4/91 vaccine regime at 1 day only or 1 + 14 days) than in the positive control groups. **Conclusions:** The heterologous combined IB Primer + 4/91 program demonstrated the most significant protective efficacy against the IBV field challenge strains compared with other vaccines in broiler chickens.

## 1. Introduction

In Egypt, various viral infections are present, causing numerous epidemics. Typically, RNA viruses cause significant viral epidemics, leading to devastating economic losses in many poultry farms [1,2,3,4]. Infectious bronchitis (IB) is considered as a substantial economic challenge globally, causing catastrophic losses by negatively influencing meat and egg productivity [5]. IB is caused by *Infectious bronchitis virus* (IBV), which is an emerging, highly contagious *Avian coronavirus*. It infects most poultry breeds across all ages, with severe symptoms in younger birds and a high mortality rate of up to 80% depending on the bird’s age, its immune status, IBV strain heterogeneity on a local basis, and subsequent co-infections with a secondary bacterial or viral pathogen [6,7,8]. Furthermore, the impact of IBV on multiple systems, such as respiratory, digestive, nervous, urinary, and reproductive systems, can complicate the management and control of the infection due to the virus’s epitheliotropic nature and the host’s susceptibility [7,8,9,10]. IBV is classified as a positive non-segmented, single-stranded RNA virus within the genus *Gammacoronavirus*, of the *Coronaviridae* family, and also designated as Avian coronavirus (ACoV) (https://ictv.global/taxonomy; accessed on 12 January 2024).

IBV is an enveloped unsegmented virus with a length of approximately 27.6 kb. The viral genome encodes four major structural protein-coding genes, including the nucleocapsid (N) protein, envelope (E), membrane (M), and spike (S) glycoprotein, as well as additional accessory proteins (3a, 3b, 5a, 4b, 4c, 5a, 5b, and 6b) [11,12,13,14]. Importantly, IBV is characterized by a high propensity for mutation, leading to the emergence of novel genotypes as well as changes in antigenic properties, tissue tropism, and disease pathogenicity [15,16]. IBV strains are clustered based on variations in the full-length sequencing of the *S1* gene [17]. Particularly, the genotypes of GI-1, GI-12, GI-13, GI-16, GI-19, and GI-23 are currently the predominant IBV genotypes in the Middle East and North Africa region [18,19].

Specifically, IBV control remains challenging, and vaccination is the most efficient strategy for managing the disease, which is regarded as the cornerstone of protection. For over two decades, there have been several classical and variant IBV vaccines originating from genotypes including GI-1, GI-12, and GI-13 which have been implemented in Egyptian poultry populations to combat the newly emerged genotype GI-23. In the Egyptian field, a variety of IBV isolates were identified and interconnected to other classic strains, such as M41, as well as pathogenic variant strains, such as D274, D1466, D3898, D3128, 793B, IS/885/00, and IS/1494/06 [20,21,22,23]. Moreover, the vaccines based on IBV strain 793B, QX-like strain [24], or IB Var II of the Middle East [25] have been established from previous pathogenic strains with better protection rates.

Subsequently, the efficacy and safety assessment of available combined IBV vaccines is crucial because the birds encounter numerous IBV variant strains simultaneously [26,27]. Additionally, the assessment of cross-protection from heterologous IBV serotypes via particular combined vaccines represents an alternative approach for managing and controlling the disease [28,29]. In addition, the vaccination protocol including combined classic and variant IBV vaccines together can significantly reduce the negative undesirable impact caused by heterologous challenges of the variant stains, supplying considerable levels of protection [30,31]. Additionally, administering Ma5 and 793B vaccines simultaneously or alternately in Europe produces significant protection against heterologous IBV strains, including D1466 or QX. Nonetheless, homologous-based vaccines frequently show a good protection level against IBV infection [32,33].

Recently, in Egypt, the field circumstances confirmed the circulating genotype (GI-23) lineage that is the predominant IBV genotype in chicken flocks [22,34,35]. Thus, the newly developed GI-23-based vaccines demonstrated an effective improved protection against homologous challenge [36,37]. Notably, the Egyptian IBV variant-2 strain demonstrates considerable genetic diversity comparable to other all imported vaccines with numerous substitution mutations [38,39]. Hence, this study targets the protective efficacy of the vaccination regimens afforded by attenuated GI-23-vaccines, either alone or in combination with other vaccines such as H120, IB Primer, Egyptian IB Var II, and IB-793/B (4/91), was evaluated against the circulating IBV strain by investigating the clinical and pathological findings, IBV shedding detection, and evaluating ciliostasis activity. This current combined vaccination protocol was implemented experimentally in specific-pathogen-free (SPF) chicks at either 1 day of age and/or 10 days of age.

## 2. Materials and Methods

### 2.1. Ethical Statement

All experimental procedures were approved by the ethics and guidelines for animal experiments set following the New Valley Research Ethics Committee, Faculty of Veterinary Medicine, New Valley University under the reference number (04-2023-200249).

### 2.2. Vaccines and Strains of Viruses

Four commercially readily available live attenuated IBV vaccines, IB Var II (Mevac, Egypt)^®^, Massachusetts serotype vaccine (H120) (MSD Animal Health, Madison, NJ, USA)^®^, Nobilis^®^ 4/91 (IB-793B) (MSD Animal Health, Madison, NJ, USA)^®^, and IB Primer (Zoetis, Inc., Kalamazoo, MI, USA)^®^, were used in this study. All the vaccines were administered to chickens via the oculo-nasal recommended route according to the manufacturer’s guidelines. The challenge virus used was a recent variant II strain, NewValley-1-EGYIBV-GI23.3-2023 (GenBank accession no.: PQ093637). The challenge virus was propagated and titrated using the method described by Reed and Muench using the egg infective dose at 50% (EID_50_) approach [40,41].

### 2.3. Experimental Design

First, healthy one-day-old SPF chicks (n = 150) were purchased (Kom-Osheim farm, Fayoum governorate, Egypt). The chicks were clustered into 10 groups (15 chicks per group) within separate isolators under strict sanitary conditions. During the entire experiment, all tested chickens were provided with feed and water ad libitum. The group designations including vaccination regimes separately and simultaneously at 1 and 14 days of age are shown in Table 1. Chicks were challenged with 10^6^ EID_50_/0.2 mL/chick with the full spike sequenced IBV NewValley-1-EGYIBV-GI23.3-2023 strain, collected from trachea, kidney, and lung tissues of the New Valley Governorate (GenBank accession no.: PQ093637), via the oculo-nasal route at 28 days of age. Importantly, tracheal swabs were collected from challenged chicks at 3, 5, and 7 days post-challenge (DPC) to quantify virus shedding. The collected swabs were placed in 1 mL of ice-cold phosphate-buffered saline (PBS) and stored at −80 °C until examination using IBV real-time RT–PCR. Particularly, at 7 DPC, the ciliary activity of the birds post-challenge was assessed, where 5 chicks/group were euthanized to evaluate the vaccine protection. All birds were monitored twice daily by recording clinical symptoms, mortality rates, and gross pathologic lesion detection in the euthanized and dead birds post-challenge. Additionally, the trachea, proventriculus, bursa, and kidneys were gathered from 3 chicks/group at 7 DPC for histopathological investigation [39,42]. The non-vaccinated/challenged and non-vaccinated/non-challenged chicks (Groups 9 and 10) were maintained as controls throughout the experiment.

### 2.4. Evaluation of Tracheal Ciliostasis

Ciliary activity evaluation was conducted by a described method [43,44]. Specifically, three sections of each trachea, from the upper, middle, and lower portions, were analyzed, each about 1 mm thick. These sections were then placed in a mixture containing minimum essential medium (MEM) with 10% fetal bovine serum in a Petri dish. Afterwards, these sections were observed under an inverted microscope to record the integrity and ciliary motion of the epithelial cells. Each three individual birds were scored in each group (Table 2), and the average ciliostasis and protection scores for each group were calculated using the following formula:

$$\left[1-\left(\frac{Mean\;ciliostasis\;score\;for\;the\;vaccinated\;challenged\;group}{Mean\;ciliostasis\;score\;for\;the\;corresponding\;non-vaccinated\;challenged\;group}\right)\right]\times 100\%$$

### 2.5. IBV Shedding Titers

Particularly, the viral RNA was extracted from the collected swabs by a Bioflux©R viral RNA Mini Spin column kit (Bioflux, Hangzhou, China) and a QIAamp Viral RNA Mini Kit (Qiagen, Hilden, Germany) following the manufacturers’ instructions. Then, a Verso 1-Step quantitative real-time reverse transcriptase PCR (qRT-PCR) Kit (Thermo Scientific, Waltham, MA, USA) was used to quantify the most conserved region of the *S1* gene of IBV with a QuantiTect^®^ probe RT-PCR kit (Qiagen, Hilden, Germany), with specific oligonucleotide primers. The forward primer is IBV-VAR II-F+ 5’-CAA TGG TCC CCG TTT GTG-3’, at 1128–1145 base pairs (bp), the reverse primer is IBV-VAR II-R- 5’-GTC TAG GAT GGC TAA ACC AC-3’, at 1385–1404 bp, and the probe is IBV-VAR II-pro+ (HEX) 5’-CCA GGA ATG AAC CAC TTG TGT TAA CTC-3’ (TAMRA), at 1235–1261 bp [45]. The reference strains included IB Var II (Mevac) vaccine, and IBV (4/91) vaccine, and Nobilis. The IBV shedding titers were subsequently determined through a standard curve generation using titrated viruses inoculated in specific pathogen-free embryonated chicken eggs (SPF-ECEs), and the shedding titers were assessed using interpolation to distinguish obviously between the vaccinal and challenge IBV strains [46]. Consequently, data is expressed as the number of IBV RNA copies (log_10_) per mL.

### 2.6. Pathological Examination

Notably, the trachea, proventriculus, bursa, and kidney of three randomly selected infected birds were collected for necropsy specimen examination at 7 DPC. The examined specimens were fixed in 10% buffered formalin, processed routinely, embedded in paraffin, cut into sections of 5 µm thickness, and finally stained with hematoxylin and eosin (H&E) for histopathological analysis using the light microscope [47]. Briefly, lesion scoring (Table 3) was performed to assess the deciliation and degeneration of tracheal epithelium, heterophil and lymphocyte infiltration, glandular epithelium hyperplasia, decreased mucous cells, and hemorrhage. In addition, kidney tissue lesions, such as tubular degeneration, renal nephrosis and necrosis, epithelial deciliation, epithelial hyperplasia, presence of renal casts, infiltration by heterophil and lymphoid cells, lymphoid nodules, and fibroblastic proliferation, were additionally scored as follows: 0 = normal, 1 = mild lesions, 2 = moderate lesions, 3 = severe lesions, and 4 = severe lesions [48,49].

### 2.7. Statistical Analysis

The descriptive analyses were reported as mean and standard deviations (±SD). Differences among groups in histopathological and ciliostasis scores, along with IBV tracheal shedding titers, were analyzed using the non-parametric Kruskal–Wallis test after assessing distribution normality with the Shapiro–Wilk normality test. After that, Tukey’s honest significant difference (Tukey HSD) post hoc test was used to assess pairwise comparisons and identify which specific pairs of group means are significantly different. Furthermore, the results of histopathological and ciliostasis scores were illustrating using the “ggplot2” package. For all analyses, *p*-value ≤0.05 was considered statistically significant.

## 3. Results

### 3.1. Genetic Similarity

The IB Primer vaccine seed strain showed a genetic relatedness to the EGYIBV-GI-23.3 isolate with amino acid and nucleotide identities percentages of 81–87%. Meanwhile, the IB Var II vaccine was closely genetically similar to EGYIBV-GI-23.3, with a nucleotide identity percentage of 90% and an amino acid identity percentage of 86% (Table 4).

### 3.2. Clinical Observations

Concerning the non-vaccinated challenged group, typical clinical symptoms were observed, including respiratory distress, huddling, increased water consumption, feather ruffling, and mild watery diarrhea, which manifested as early as 3 DPC till 7 DPC. In the negative control group, no obvious clinical symptoms attributed to IBV infection were observed. However, some birds in some vaccinated groups exhibited mild signs of depression. Notably, no IBV was detected in the birds vaccinated with IB Primer + 4/91 vaccines at 1 day or 1 and 14 days of age, demonstrating this effectiveness. Additionally, no mortality cases were observed throughout the experimental trial period among chickens in all vaccinated and control groups.

### 3.3. Tracheal Post-Challenge Ciliary Activity

At 7 DPC, the tracheal ciliostasis activity scores were assessed to ascertain the safety and protective efficacy level of various IBV vaccination regimens. Surprisingly, post hoc analysis using Tukey’s HSD test demonstrated that vaccinated groups receiving combined IB Primer + 4/91 vaccines at 1 day (G-1) and/or 1 day + 14 days of age (G-5) showed a noticeably lower ciliostasis score (1.4 ± 0.08 and 1.3 ± 0.14, respectively) with an effectively ciliary protection percentage ranging from 65–68% (Table 5), which was significantly different (*p* < 0.05) in comparison to the other vaccinated groups. Likewise, the IB Primer + IB Var II (Mevac) vaccinated groups exhibited slightly low ciliostasis scores among groups (1.5 ± 0.08, *p* < 0.05) (“Supplementary File Table S1”, Table 5). Furthermore, the vaccinated groups receiving the H120 + 4/91 vaccine regime at 1 day + 14 days of age showed a moderate ciliostasis score of 1.9 ± 0.08 with a protection percentage of 53%, with no significant difference compared to the positive and negative control groups. Meanwhile, the H120 + IB Var II vaccine regime at 1 + 14 days of age (G-8) exhibited the highest ciliostasis score of 2.75 ± 0.12 with the lowest protection of 31% among all vaccinated groups. Moreover, the last group (negative control) displayed a high mean score of 0.7 ± 0.08; 83% protection; maintaining intact cilia. The positive control (non-vaccinated, challenged) group displayed complete ciliostasis (4.0 ± 0.49; 0% protection). Finally, there was variation in ciliary activity scores in the groups vaccinated with H120 + 4/91 and H120 + IB Var II at (1 day and/or 1 day + 14 days) observed between 1.9 ± 0.08 and 2.75 ± 0.12 with a low protection level ranging from 31–53% (Table 5).

### 3.4. Quantification of IBV Shedding Using qRT–PCR

qRT-PCR detection was performed by testing in duplicate serial tenfold dilutions of the IBV variant II, and standard curves were produced after adjusting them to 10^7^ 50% egg infectious dose (EID_50_)/mL. The standard curves had a coefficient of determination (R^2^) value of 0.94. Significantly, the shedding titers were checked at 3, 5, and 7 DPC in all groups receiving multiple vaccine protocols. The shedding titers at 3, 5, and 7 DPC showed a significant viral load reduction across all existing vaccinated groups (*p*-value < 0.05) compared to the positive control one at all time points, except for another group that was vaccinated with the H120 + IB Var II vaccine regime (1 + 14 days old), which showed no significant difference from the positive challenge group at both 5 DPC and 7 DPC. Statistically, at 3 and 5 DPC, the highest shedding titers were recorded in the positive control, followed by the H120 + IB Var II (1 day and/or 1 day + 14 days) and H120 + 4/91 (1 day) groups. Significantly, lower titers were observed in the IB Primer + IB Var II (1 day) and H120 + 4/91 (1 day + 14 days) groups. Conversely, a marked and significant reduction was observed in group 5, which received the IB Primer + 4/91 vaccine regime at 1 day and 14 days. However, the reduction remained significantly lower (*p*-value < 0.05) than that observed in the groups vaccinated with the H120 + IB Var II vaccine at 1 day and H120 + IB Var II vaccine 1–14 days at all time points. At 7 DPC, viral shedding decreased across all groups but remained highest in the H120 + IB Var II (1 day and/or 1 day + 14 days) and positive control groups. Regarding the number of shedders, groups 4 and 8 that received the H120 + IB Var II vaccine regime exhibited elevated numbers of virus shedders at every time point compared to all other groups (Table 6).

### 3.5. Histopathological Examination

**Figure 1:**
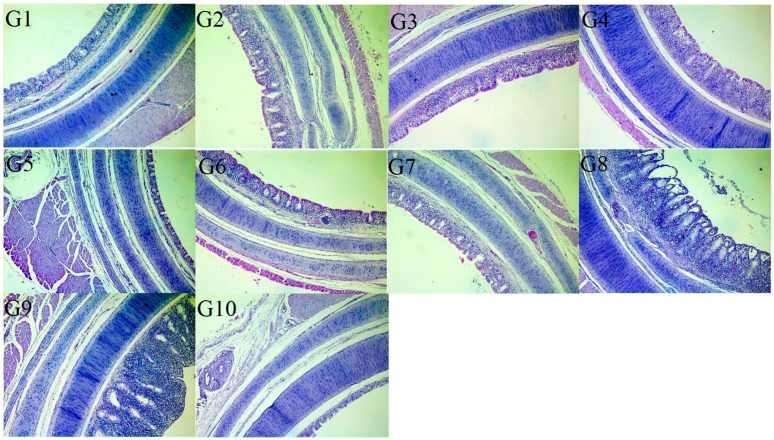
The histopathological findings of the tracheas of the vaccinated challenged groups at 7 DPC showed mild epithelial hyperplasia and metaplasia with cyst formation in G-5, G-1, and G-6. Moderate epithelial hyperplasia and metaplasia with cyst formation appeared in G-7, G-3, and G-2. Severe epithelial hyperplasia, desquamation and leukocytic infiltration (G-4 and G-8). The Positive control group (unvaccinated challenged group) (G-9) showed severe hyperplasia of the lining epithelium, cyst formation, and mucosal thickening associated with severe leucocytic infiltration. Negative control group (unvaccinated unchallenged group) (G-10) trachea showed normal tissue histology (H&E, X100).

Concerning macroscopic examination (Supplementary File S2), the tracheas of G-10 (negative control) were apparently normal, whereas those of G-9 (positive control) showed congestion with clear to turbid mucus in the lumen. The tracheas in groups G-8 and G-4 were congested with mucus. Little mucus was seen in the trachea of the G-7, G-3, and G-2 groups. Group G-5, G-1, and G-6 tracheas showed the mildest gross lesions similar to the control group. Microscopically, the tracheas of G-10 (negative control) revealed an apparently normal tissue histology. Meanwhile, in G-9 (positive control), the surface tracheal epithelium revealed loss of cilia, hyperplasia, and increased mucosal glands with degeneration or necrotic changes beside leukocytic infiltration, mainly heterophils and lymphocytes, in the mucosa and submucosa. Also, the tracheal serosa showed moderate congestion of blood vessels with thickened walls, perivascular edema, and few leukocytic infiltrations. Histopathological assessment at 7 DPC revealed that the tracheas from birds receiving the IB Primer + 4/91 vaccine regime at 1 and 14 days (G-8) and those vaccinated with the H120 + IB Var II vaccine (at 1D) (G-4) showed the most severe lesions among other groups vaccinated with various regimes. Meanwhile, G-7, G-3, and G-2 showed moderate lesion scores representing mild epithelial hyperplasia, metaplasia, and cyst formation in addition to leukocytic infiltration in G-3. The tracheal lesions in G-5, G-1, and G-6 were the mildest of the abovementioned lesions (Figure 1).

**Figure 2:**
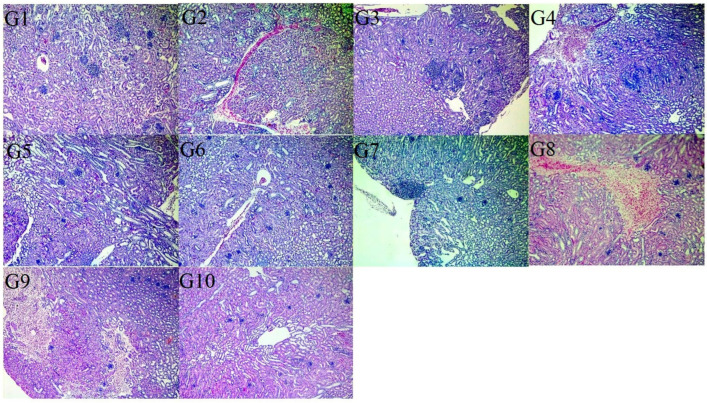
The histopathological findings of the kidneys of the vaccinated challenged groups at 7 DPC. Variable degrees of hemorrhage in the G-4, G-8, and G-9 groups. Lymphocytic aggregation in the G-2, G-3, and G-7 groups. Congested blood vessels in G-2. Glomerular hypercellularity was observed in G-5. The positive control group (unvaccinated challenged group) (G-9) showed necrosis replaced by RBCs and lymphocytic infiltration. Negative control group (unvaccinated unchallenged group) (G-10) kidneys showed normal tissue histology (H&E, X100).

Regarding macroscopic examination of the kidneys, those of G-10 (negative control) were apparently normal, while those of G-9 (positive control) showed enlargement and varied in color from dark red to pale. Microscopically, the kidneys of G-10 (negative control) revealed no pathological lesions, while those of G-9 (positive control) showed intertubular lymphocytic aggregations replacing some necrotic renal tubules with pressure atrophy of the adjacent degenerated tubules. Extravasation of erythrocytes was detected in the renal medulla in some cases. Some glomeruli showed hypercellularity with a thickened basement membrane, whereas others showed complete obliteration of the glomerular space and hyalinization of the glomerular tuft. Hydropic degeneration or necrosis of the epithelial cells of other renal tubules, represented by pyknosis, karyorehexis, or karyolysis of their nuclei, was detected. The ureter revealed leucocytic infiltration of lymphocytes in the lamina propria. The kidney of G-1, G-5, and G-6 revealed a mild degree of lymphocytic aggregation and leukocyte infiltration, mild congestion of blood vessels, and few scattered RBCs among renal tubules. Groups G-2, G-3, and G-7 showed similar lesions and, in addition G-7 revealed mild to moderate hemorrhage. However, G-8 and G-4 kidneys showed moderate to severe hemorrhage in addition to glomerular hypercellularity and degeneration of the tubular epithelium (Figure 2).

**Figure 3:**
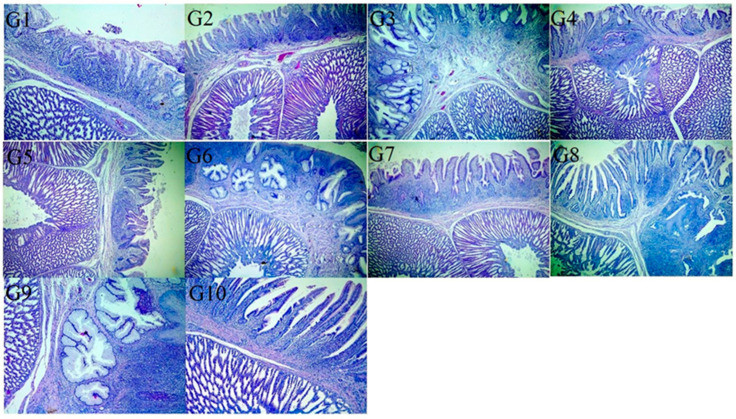
Proventriculus of vaccinated challenged groups at 7 DPC showed mild leukocytic cell infiltration and cyst formation in groups G-1, G-5, and G-6. Groups G-2, G-3, and G-7 exhibited moderate lymphocytic infiltration, whereas G-4 and G-8 showed moderate lymphocytic infiltration with cyst formation in the submucosa. The positive control group (unvaccinated challenged group) (G-9) showed severe leukocytic infiltration and epithelial cyst formation in the submucosa. Negative control group (unvaccinated unchallenged group) (G-10) proventriculus showed nearly normal mucosal and submucosal layers (H&E, X100).

**Figure 4:**
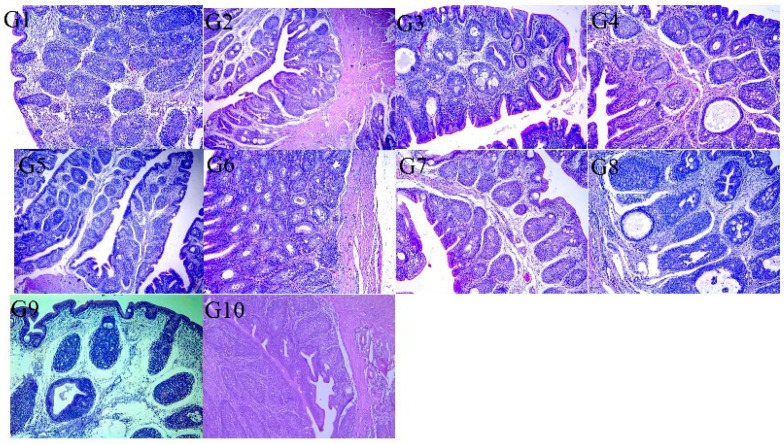
The histopathological findings of the bursa of the vaccinated challenged groups at 7 DPC. Variable degrees of lymphocyte thickening depletion, interfollicular septal widening, and epithelial cell cyst formation in groups G-1, G-2, G-3, G-5, G-6, and G-7 and moderate lesions in groups G-4 and G-8. The positive control group (unvaccinated challenged group) (G-9) showed severe depletion of follicular lymphocytes, thickening of interfollicular septa, necrosis of lymphocytes replaced by cyst formation. Negative control group (unvaccinated unchallenged group) (G10) bursa showed normal tissue histology (H&E, X100).

Macroscopic investigation of the proventriculus revealed that the G-10 (negative control) group was apparently normal, whereas that of the G-9 (positive control) group was slightly enlarged. Groups G-1, G-5, G-6, G-2, G-3, and G-7 showed milder lesions than groups G-4 and G-8. Microscopically, the epithelium lining of the proventriculus mucosa of some chickens showed hyperplasia and desquamation within its lumen. The mucosa and submucosal glands were necrotic and replaced by mononuclear cell infiltration. The compound glands of the submucosa revealed hyperplastic and vacuolated epithelium with connective tissue septae thickening by edema. The muscular layer showed hyaline degeneration, and the serosal blood vessels were congested, revealing thickening and vacuolation of the tunica media together with endotheliosis. In the other groups, the proventriculi showed mild (G-1, G-5, and G-6) to moderate (G-2, G-3, and G-7) lymphocytic infiltration, whereas those of G-4 and G-8 showed cyst formation in the submucosa (Figure 3). Macroscopically, the bursa of G-10 (negative control) was apparently normal, whereas that of G-9 (positive control) was slightly atrophied in the majority of birds. Finally, microscopically, the bursa of G-10 (negative control) revealed apparently normal tissue histology, whereas in G-9 (positive control), the epithelium covering the bursa showed vacuolization and vesicle formation, whereas others revealed hyperplasia. Some lymphoid follicles showed depletion and necrosis or cyst formation in the medullary zones with edematous fibrous tissue causing interfollicular expansion. Congested blood vessels in the interfollicular and subepithelial tissues were also observed. The serosal layer was thickened. Variable degrees of the abovementioned lesions were observed in the vaccinated groups (G-1, G-2, G-3, G-5, G-6, and G-7) moderate lesions in groups G-4 and G-8 (Figure 4).

## 4. Discussion

IBV is a significant viral pathogen that poses a major risk to the global poultry industry. In Egypt, IBV is highly prevalent in both vaccinated and unvaccinated flocks, leading to substantial economic losses due to the numerous circulating IBV serotypes and genotypes. Serious IBV outbreaks continue to emerge recurrently in various regions of Egypt despite comprehensive vaccination efforts with various IBV vaccines. The rising outbreak incidence of vaccinated poultry populations underscores the inadequate level of protection provided by these vaccines, making disease control more challenging [50,51]. Additionally, the administration of live attenuated vaccines to broilers plays a crucial role in the vaccination protocol against IBV, depending on the stimulation of the mucosal immunity in order to prevent clinical manifestations and mortality [26,27]. Recently, in Egypt, IBV’s continuous evolution has negatively influenced the effectiveness of licensed vaccines, as no protectotype has been identified against the IBV-GI-23 lineage circulating in the Middle Eastern region [17]. Interestingly, this study targets the efficacy of various combinations of homologous and heterologous live IBV vaccines, including classical and/or variant serotypes, compared in SPF chickens to assess the protection level gained by various vaccination protocols against the IBV-GI-23 lineage.

The current data provides intensive interesting findings on IBV vaccination programs against wild-type GI-23.3 in poultry flocks in Egypt. In this experiment, broiler chicks were used as they are routinely given various IBV vaccines simultaneously in the hatchery, and accurate information is required to know which would be relevant to poultry producers. Collectively, there were no obvious clinical symptoms associated with IBV infection, except for a few birds in some vaccinated groups that showed mild manifestations and the positive control group that showed noticeable clinical symptoms as early as 3 DPC till 7 DPC. These current findings are in accordance with those from earlier studies [39,52,53,54]. Specifically, G-5 and G-1 vaccinated with the IB Primer + 4/91 regime at 1 day or at 1 and 14 days of age exhibited the absence of IBV symptoms, confirming the effectiveness of the treatment. Clinically, no cases of mortality were observed among chickens associated with applying multiple IBV vaccines in all vaccinated and control groups. This is because these birds held in isolation units are maintained under the best environmental circumstances. Multiple previous studies have demonstrated no mortalities in commercial and SPF chicks challenged with wild strains, including EG/1212B (GI-23.2.2) and IS/885/00 (GI-23.2.1) [25,26,55]. Concerning IBV control, traditional M41 and H120 vaccines have been widely applied, despite the high diversity of IBV genomes, which lowers the vaccination protection rate of newly emerged strains. In addition, IBV vaccination using a single vaccine can only provide restricted protection from different serotypes/genotypes, resulting in the emergence of new IBV variant strains and posing considerable challenges in disease elimination and control [56,57,58]. However, numerous alternative options, such as recombinant vaccine [59], nanoparticle-based vaccine [60], and epitope-based vaccines [61], have been used to provide extensive protection against variant IBV strains. Thus, the proper and effective selection of vaccines and vaccination regimes is highly crucial to the control of IBV in the field [31]. As depicted in our results, the vaccinated groups combining IB Primer + 4/91 (G-1, G-5) programs at 1 day or at 1 + 14 days of age revealed the best protection (65, 68%, respectively) with a visibly lower ciliostasis score (1.3 ± 0.14, 1.4 ± 0.08, respectively), followed by the group vaccinated with the IB Primer + IB Var II regime (1 + 14 days) with a score of (1.5 ± 0.08) and 63% protection. This current protection level can be attributed to a broad-spectrum immunological response triggered by the usage of heterologous combined IBV vaccines. However, the IB Var II vaccine, a locally manufactured vaccine, showed homology with the challenge strain (EGYIBV-GI-23.3) with amino acid and nucleotide identity percentages of 86–90%. Its protective level was was considerably lower than that of IB Primer vaccine. This may be attributed to its adverse effects, including the post-vaccinal reaction of the vaccine and its increasing effect of ciliary activity on tracheal tissue. From previous results, the IB Primer vaccine has already been proven to broaden protection against various field strains of IBV as it is a heterologous protectotype vaccine containing both classic and variant strains. Nevertheless, it is essential to consider the impact of attenuated vaccines on tracheal ciliostasis when evaluating IBV vaccine efficacy [62] as an attenuated vaccine can produce a definite degree of ciliostasis that varies with the attenuation degree [26,27,63]. Meanwhile, 50% protection was reported in the groups vaccinated with the IB Primer + IB Var II (G-3) (day 1) regime with a ciliary score of (2 ± 0.01). This subsequent finding further highlights the significance of the IBV vaccine homology to the most prevalent field strains in order to achieve the optimum protective efficacy. Similarly, the groups vaccinated with the H120 + IB Var II regime at 1 day (G-4) and 1 + 14 days of age (G-8) revealed higher ciliostasis scores (2.5 ± 0.08, 2.77 ± 0.12) with the lowest protection rate (38%, 31%, respectively). This previous observation did not align with other reports [53] that reported that the H120 + IB Var II vaccine regimen administered at day 1 and IB Var II at day 14 achieved the lowest ciliostasis score with the highest protection rate (89.58%). Furthermore, several studies confirmed our current observations as these studies evaluate the vaccine efficacy (H120, Connecticut, Mass-type, IB-793B (4/91), CR88, and D274) either individually or in combination against GI-23 challenge, showing low protection levels (30–77%) [55,64,65]. From the abovementioned findings, it is clear that genotype-specific vaccine usage in vaccination regimens can improve IBV vaccination efficacy against characteristic genotypes. This is because the existence of genetic variations within the *S* gene of similar serotypes may lead to varied impacts on IBV vaccine effectiveness. In particular, a critical divergence in the efficacy of IBV vaccine regimes may also be attributed to the experimental chicks’ origin. Interestingly, the ability to decrease IBV multiplication in the vaccinated chickens is an essential guide for an efficient vaccination schedule, and it has subsequently been evaluated by qRT-PCR that quantifies the viral RNA load in tracheal swab specimens [25,39]. Currently, all vaccinated groups exhibited variable viral load reductions. More importantly, a noticeable reduction in viral load was observed clearly in the groups G-1 and G-5 receiving the IB Primer + 4/91 program at 1 day and 1 + 14 days of age compared with the H120 + IB Var II vaccine (1D) and H120 + IB Var II vaccine (1D–14D) at every time point. These subsequent results do not closely agree with [53] who stated that a noteworthy reduction in viral load was noticed in various vaccine programs such as H120 + IB Var II at 1 day and IB Var II at 14 days, IB Var II at 1 day and H120 at 14 days, and H120 at 1 day and IB Var II at 14 days. Significantly, a strong correlation was established between the virulence of the vaccinal strain and the cellular and local immune responses [66]. This correlation has a significant impact on the suitability of such regimes applied in Egypt considering the exposure of vaccinated birds to several respiratory viral and/or bacterial pathogens that interact with IBV [67,68]. Parallel to our results, [43] mentioned that wider protection against heterologous IBV types could be accomplished by administering two different IBV vaccines either simultaneously or as a prime and boost vaccination.

Furthermore, histopathological investigation was performed to compare IBV-induced lesions in the trachea, kidney, bursa of Fabricius, and proventriculus. Our results revealed significant lesion scores in the IBV-infected control group (G-9) at 7 DPC. These tracheal lesions include desquamated epithelium in the tracheal lumen, epithelial degeneration, loss of cilia, congestion of blood vessels, and lymphocytic infiltration. In agreement with these results, a previous study observed the tracheal histomorphometry in terms of mucosal thickness and lymphocyte infiltration in the trachea [69]. However, another study on 65-week-old hens and cockerels, challenged with T and N1/88 Australian IBV strains, reported that the cockerels had edema in the mucosa and alveolar mucous gland hypertrophy [53,70]. In the case of kidney lesions, there were congestion, hemorrhages, renal tubule degeneration, and interstitial nephritis. Specifically, heterologous IBV vaccine regimes (G-5, G-1, G-6) can reduce the tracheal and kidney lesion scores with high protection percentages and relatively normal structures. These subsequent results are in parallel with [53,54] who reported that the application of heterologous IBV vaccines demonstrated a significant protective efficacy against the Thai QX-like virus. Additionally, the proventriculus showed hyperplasia of mucosal epithelium lining with desquamation, and sometimes mononuclear cell infiltration in the mucosa, submucosa, and interglandular tissue, replacing the necrotic tissue. These histologic findings are in accordance with [71]. Moreover, the infected bursa exhibited an atrophy of lymphoid follicles (small-sized follicles), lymphocyte depletion, and increased interfollicular and interseptal space. The results were similar to those reported by [72].

## 5. Conclusions

This study emphasizes the crucial role of efficient vaccination strategy in reducing the clinical symptom severity and mortalities associated with IBV. The usage of heterologous IBV vaccines, including the combined IB Primer + 4/91 program either at 1 day or at 1 + 14 days of age, revealed a significant protective efficacy against the field challenge of local IBV-GI-23.3. Additionally, the live attenuated vaccine efficacy is closely related to the genetic similarity between the vaccine and IBV field isolates. Likewise, vaccine field efficacy might be influenced by additional factors, including maternal immunity interference, co-infections with other pathogens, improper vaccine handling or administration, and management practices. This proactive vaccination strategy provides efficient clinical protection, helping to mitigate the IB infection impact, and minimizes its economic losses in the local poultry in Egypt. Lastly, further cross-protection studies are necessary to determine the vaccinal strains’ effect on the tracheal ciliostasis activity for vaccine assessment and appropriate design of vaccination regimes, considering field complications with other respiratory infections in Egypt.

## Figures and Tables

**Table 1:** Experimental design of IBV vaccination regimes separately and simultaneously at 1 and 14 days of age.

Group	Vaccination Regime	Number of Chicks	Age at Vaccination		Number of Challenged Chicks
			1 Day	14 Days	
1	IB Primer + 4/91	15 in each group	IB Primer + 4/91	-	15
2	H120 + 4/91		H120 + 4/91	-	15
3	IB Primer + IB Var II (Mevac)		IB Primer + IB Var II (Mevac)	-	15
4	H120 + IB Var II (Mevac)		H120 + IB Var II (Mevac)	-	15
5	IB Primer + 4/91		IB Primer	4/91	15
6	IB Primer + IB Var II (Mevac)		IB Primer	IB Var II (Mevac)	15
7	H120 + 4/91		H120	4/91	15
8	H120 + IB Var II (Mevac)		H120	IB Var II (Mevac)	15
9	Positive control		Non-vaccinated challenged group		15
10	Negative control		Non-vaccinated and non-challenged group		15

**Table 2:** Ciliary activity was scored as follows.

Score	Ciliary Activity
0	100% ciliary activity, all cilia beating (complete protection)
1	75 percent of cilia beating
2	50 percent of cilia beating
3	25 percent of cilia beating
4	0 percent ciliary activity, non-beating cilia (complete lack of protection)

**Table 3:** Tracheal tissue lesions scoring.

Score	Trachea (% of Wall Affected)
0	No change
1	25%
2	26–50%
3	51–75%
4	76–100%

**Table 4:** Nucleotide and amino acid identities of NewValley-1-EGYIBV-GI23.3-2023 compared with those of other vaccines.

Sample ID		NewValley-1-EGYIBV-GI23.3	IBV-H120-GI-1	IBV-D274, GI-12	IBV-4/91-GI-13	IBV-GI-23-EG/1212B-2012	
Nucleotide identity (%)							
1	PQ093637: NewValley-1-EGYIBV-GI-23.3-2023	ID	84%	87%	86%	90%	1
2	IBV- H120-GI-1	75%	ID	85%	83%	82%	2
3	IBV- D274, GI-12	81%	78%	ID	86%	86%	3
4	IBV- 4/91-GI-13 (4/91 primer vaccine)	78%	75%	79%	ID	83%	4
5	IBV-GI-23-EG/1212B-2012 (IB Var II vaccine)	86%	74%	82%	75%	ID	5
Amino acid identity (%)							

**Table 5:** Mean ciliostasis scores with standard deviation (±SD) and protection percentage using various vaccination regimens following field strain IBV-GI-23.3 challenge.

Group	Vaccination	Ciliary Score (±SD)	Ciliary Protection
**G1 ^a^**	**IB Primer + 4/91 (1D)**	**1.4 ± 0.08**	**65%**
G2 ^b^	H120 + 4/91 (1D)	2.1 ± 0.07	48%
G3 ^b^	IB Primer + IB Var II (1D)	2 ± 0.01	50%
G4 ^c^	H120 + IB Var II (1D)	2.5 ± 0.08	38%
**G5 ^a^**	**IB Primer + 4/91 (1D-14D)**	**1.3 ± 0.14**	**68%**
**G6 ^ab^**	**IB Primer + IB Var II (1D-14D)**	**1.5 ± 0.08**	**63%**
G7 ^bc^	H120 + 4/91 (1D-14D)	1.9 ± 0.08	53%
G8 ^d^	H120 + IB Var II (1D-14D)	2.75 ± 0.12	31%
G9 ^e^	Positive control	4 ± 0.49	0%
G10 ^f^	Negative control	0.7 ± 0.08	83%

1D is one day, 14D is fourteen days. H120 vaccine is the classical GI-1 strain. IB Var II vaccine refers to the variant II GI-23 strain. Positive control refers to the unvaccinated challenged group, and negative control denotes the unvaccinated unchallenged group. The treated groups showed significantly lower ciliostasis scores compared to the positive control group. Bold: The observed highest protection was presented in the group vaccinated with IB Primer + 4/91 (1 day and/or 14 days) (G-1, G-5), and then IB Primer + IB Var II (G-6) at 1 day + 14 days, while the lowest protection was detected in the group vaccinated with H120 + IB Var II (G-8) (1 day + 14 days). Means with different letters (a–f) in the same column differ significantly (*p* < 0.05) according to Tukey’s HSD post hoc test.

**Table 6:** Mean values with standard deviation (±SD) of tracheal shedding quantification (Log_10_ EID_50_/_mL_) in eight groups receiving various vaccination protocols following IBV-GI-23.3 challenge.

Groups and Vaccination Regime (1 Day Old)	Virus Shedding Titers (EID_50_/_mL_) at Specific Time Points		
	3 DPC	5 DPC	7 DPC
G1: IB Primer + 4/91 ^a^	3.53 ± 0.21	1.81 ± 0.23	1.11 ± 0.18
G2: H120 + 4/91 ^b^	5.67 ± 0.45	4.64 ± 0.40	3.74 ± 0.36
G3: IB Primer + IB Var II ^b^	4.93 ± 0.36	4.53 ± 0.31	3.45 ± 0.27
G4: H120 + IB Primer Var II ^c^	5.87 ± 0.37	4.76 ± 0.31	4.16 ± 0.29
**Groups and vaccination** **regime (1 + 14 days old)**			
**G5: IB Primer + 4/91 ^a^**	**3.15 ± 0.13**	**1.65 ± 0.12**	**0.9 ± 0.10**
G6: IB Primer + IB Var II ^ab^	3.72 ± 0.18	2.12 ± 0.18	1.52 ± 0.14
G7: H120 + 4/91 ^bc^	3.98 ± 0.23	3.76 ± 0.21	3.03 ± 0.20
G8: H120 + IB Var II ^d^	6.04 ± 0.31	4.81 ± 0.28	4.82 ± 0.24
G9: Positive control ^e^	6.16 ± 0.13	5.49 ± 0.60	4.85 ± 0.33
G10: Negative control ^f^	0	0	0

Group name explanation: IB Primer: GI-1,12 vaccine strain, IB Var II: variant II GI-23 vaccine strain, H120: classical GI-1 vaccine strain, 4/91: GI-23, 793/B serotype, positive control: unvaccinated challenged group. There was a significant reduction in the viral load (*p*-value < 0.05) detected in all vaccinated groups compared to the positive control group at all time points, except for H120 + IB Var II (1 + 14 days) at 5 DPC and 7 DPC. EID_50_, 50% egg infective dose, DPC, days post-challenge. ^a bold^ Group 5 receiving IB Primer + 4/91 vaccine regime at 1 and 14 days (*p*-value <0.05) compared to other groups vaccinated with H120 + IB Var II vaccine (at 1D) and H120 + IB Var II vaccine (at 1D–14D) at all time points. Means with different letters (a–f) in the same column differ significantly (*p* < 0.05) according to Tukey’s HSD post hoc test.

## Data Availability

Data is available upon request.

## Supplementary Materials

The following supporting information can be downloaded at: https://www.mdpi.com/article/10.3390/vaccines13111132/s1, Table S1: The values and mean ciliostasis scores using various vaccination regimens following field strain IBV-GI-23.3 challenge. Legend: 1D is one day, 14 D are fourteen days. H120 vaccine is the classical GI-1 strain. VAR II vaccine refers to the variant II GI-23 strain. Positive control refers to the unvaccinated challenged group, and negative control denotes the unvaccinated unchallenged group. The treated chicks groups showed significantly lower ciliostasis scores comparable to the positive control group. Bold: The observed highest protection was presented in the group vaccinated with IB primer-4/91 (1 day and/or 14 days) (GR 1, 5), and then IB primer-VAR II (GR 6) at (1 day + 14 days); Table S2: Histopathological lesion scores 7DPC. Legend: 0 = Normal, 1 = mild, 2 = moderate, 3 = severe, 4 = very severe.
