# Supplementary material for: Successive Efficacy Evaluation of Various Commercial Live-Attenuated Avian coronavirus Vaccination Schedules Against a Local GI-23.3 Challenge in SPF Broilers
Source: Vaccines (Basel). 2025 Nov 2;13(11):1132. doi: 10.3390/vaccines13111132 (PMC12656769; doi:10.3390/vaccines13111132)
Supplement: Supplementary file 1 [file vaccines-13-01132-s001.zip › vaccines-3875773-supplementary.pdf]

Table S1. The values and mean ciliostasis scores using various vaccination regimens following field strain IBV-GI-23.3 challenge.

fGroup	Vaccination	values	mean
**GR-1**	**IB Primer-4/91 (1D)**	**1.4**	**1.40**
		1.5	
		1.3	
GR-2	H120-4/91 (1D)	2.1	2.11
		2.2	
		2.03	
GR-3	IB Primer -VAR II (1D)	2	2.01
		2.02	
		2.01	
GR-4	H120-VAR II (1D)	2.5	2.5
		2.4	
		2.6	
**GR-5**	IB **Primer -4/91 (1D-14D)**	**1.2**	**1.3**
		**1.2**	
		**1.5**	
**GR-6**	IB **Primer-VAR II (1D-14D)**	**1.6**	**1.5**
		**1.4**	
		**1.5**	
GR-7	H120-4/91 (1D-14D)	1.9	1.9
		2	
		1.8	
GR-8	H120-VAR II (1D-14D)	2.8	2.77
		2.9	
		2.6	
GR-9	Positive control	4.6	4.03
		4.1	
		3.4	
GR-10	Negative control	0.8	0.70
		0.7	

		0.6	

1D is one day, 14 D are fourteen days. H120 vaccine is the classical GI-1 strain. VAR II vaccine refers to the variant II GI-23 strain. Positive control refers to the unvaccinated challenged group, and negative control denotes the unvaccinated unchallenged group. The treated chicks groups showed significantly lower ciliostasis scores comparable to the positive control group. Bold: The observed highest protection was presented in the group vaccinated with IB primer-4/91 (1 day and/or 14 days) (GR 1, 5), and then IB primer-VAR II (GR 6) at (1 day + 14 days).

TableS2 Histopathological lesion score **:** s 7DPC.

Lesion	Group No.									
	G1	G2	G3	G4	G5	G6	G7	G8	G9	G10
Trachea lesions	1	2	2	3	1	1	2	3	3	0
Deciliation and degeneration of tracheal epithelium										
Leukocytic infiltration										
Glandular epithelium hyperplasia										
Congestion										
Kidney lesions	1	2	2	3	1	1	2	3	3	0
Hemorrage										
Degeneration of renal tubules										
Nephritis										
Congestion										
Bursa lesions	1	1	1	2	1	1	1	2	3	0
Depletion of lymphocytes										
Thickening of inter follicular septa										
Cyst formation										
Proventriculus lesions	1	2	2	2	1	1	2	2	3	0
Leuckocytic infiltration										
Cyst formation										

0=Normal, 1= mild, 2=moderate, 3-severe, 4=very severe
